# Caesarean delivery and its correlates in Northern Region of Bangladesh: application of logistic regression and cox proportional hazard model

**DOI:** 10.1186/s41043-015-0020-2

**Published:** 2015-07-31

**Authors:** Mostafizur Rahman, Asma Ahmad Shariff, Aziz Shafie, Rahmah Saaid, Rohayatimah Md. Tahir

**Affiliations:** 1 University of Rajshahi, Rajshahi, Bangladesh; 2 Centre for Foundation Studies in Science, University of Malaya, Kuala Lumpur, Malaysia; 3 Department of Geography, Faculty of Arts and Social Sciences, University of Malaya, Kuala Lumpur, Malaysia; 4 Department of Obstetrics and Gynaecology, Faculty of Medicine, University of Malaya, Kuala Lumpur, Malaysia

**Keywords:** Caesarean delivery, Risk factors, Logistic regression, Cox model, AIC, Bangladesh

## Abstract

**Background:**

Caesarean delivery (C-section) rates have been increasing dramatically in the past decades around the world. This increase has been attributed to multiple factors such as maternal, socio-demographic and institutional factors and is a burning issue of global aspect like in many developed and developing countries. Therefore, this study examines the relationship between mode of delivery and time to event with provider characteristics (i.e., covariates) respectively.

**Methods:**

The study is based on a total of 1142 delivery cases from four private and four public hospitals maternity wards. Logistic regression and Cox proportional hazard models were the statistical tools of the present study.

**Results:**

The logistic regression of multivariate analysis indicated that the risk of having a previous C-section, prolonged labour, higher educational level, mother age 25 years and above, lower order of birth, length of baby more than 45 cm and irregular intake of balanced diet were significantly predict for C-section. With regard to survival time, using the Cox model, fetal distress, previous C-section, mother’s age, age at marriage and order of birth were also the most independent risk factors for C-section. By the forward stepwise selection, the study reveals that the most common factors were previous C-section, mother’s age and order of birth in both analysis. As shown in the above results, the study suggests that these factors may influence the health-seeking behaviour of women.

**Conclusions:**

Findings suggest that program and policies need to address the increase rate of caesarean delivery in Northern region of Bangladesh. Also, for determinant of risk factors, the result of Akaike Information Criterion (AIC) indicated that logistic model is an efficient model.

## Background

Delivery may occur either by caesarean or non-caesarean. A multiple factors associated with safe delivery practices, ranging from demographic to socio-economic [1]. More than 70 % of the deliveries took place at home, and only 32 % birth in Bangladesh were under safe and hygienic conditions [2]. In recent years, caesarean delivery is one of the most common surgical procedures. Caesarean sections (C-section) are more common among first births (12.7 %), births in urban areas (15.9 %), and especially among births in the private sector (67.3 %), whereas the public sector was (34.6 %) [2]. The rate of C-sections is increasing in Bangladesh. In 2001, only 2.6 % of births were delivered by C-section, compared with 12.2 % in 2010 [3]. The number of caesarean delivery has also been growing in many developed and developing countries [4, 5] and this increase has not been clinically justified [6]. Over the last few years, the rates of C-section have risen substantially in many countries such as Brazil (30 %), [7] Chile (40 %), [8] USA (24.4 %) [9] and Malaysia (15.7 %) [10]. According to WHO, there is no justification for any region to have a caesarean rate higher than 10–15 %. This signifies a serious cause for concern in most of the countries in the world and due to several investigations into the reasons for the rising rates in caesarean delivery, it is now identified as emerging “global epidemic” [11, 12].

The increase in caesarean deliveries has been attributed to multiple factors ranging from maternal, socio-demographic and institutional factors. Caesarean delivery rates are known to vary widely among different population groups, with known risk factors including maternal age, [13–15] order of birth, [16] baby weight, [17] socioeconomic status, [18] high levels of maternal education, [19–23] previous c-section, [24–26] obstetric complications, [24, 28] maternal request (refers to a primary caesarean delivery performed because the mother requests this method of delivery in the absence of conventional medical or obstetrical indications) [27–29] and high income level [18, 22, 30–33]. The increase in caesarean delivery rates has also raised questions in Bangladesh like in most other countries. Though increased caesarean rates have been questioned and emphasized, for the lack of reliable administrative records, no early studies were carried out to identify the possible risk factors associated with C-section in this country. However, some related studies have been conducted in other countries. This study presents the most recent estimate of C-section delivery in northern region of Bangladesh and examines the association of reported complications around delivery as well as socio-demographic and relevant characteristics of women with C-section using data from sample survey. To investigate the significant relationship between mode of delivery (that is, caesarean or non-caesarean) and covariates (independent variables), most of the studies carried out logistic regression model [16, 18, 26, 34]. On the other hand, time (it is measured as after marriage to getting one child or previous child to current child) is one of the factors that may play an important role in C-section but it is not yet considered in other studies. From this point of view, the study also examined the relationship between time to event (that is, caesarean or non-caesarean) and covariates. In these cases, Cox’s model is considered to be the most used procedure for modelling the relationship of covariates to a survival or other censored outcomes [35]. Consequently, to obtain a more complete assessment of risk factors, this study considered time as a dependent variable and the concomitant variables (covariates) as independent variables and compared the results of empirical data analyzed by logistic regression and Cox proportional hazard model.

## Methods

### Study area

The study sample comprised of 1142 women who had delivery either by caesarean or non-caesarean delivery at four private and four public hospitals maternity wards in the northern region of Bangladesh during the period of January to March 2010. Among the 1142 delivery cases, 652 were caesarean and the remaining 490 were non-caesarean. The northern region is the part of north in Bangladesh, where the hospitals were situated. The hospitals involved in the study are Islamic bank hospital, Shapla, Rangpur and city clinic which are the private hospitals, while Rajshahi, Bogra, Rangpur and Dinajpur medical college hospitals are expressed as public hospitals. Also, the terms private and public patients refer to respondents, who were admitted in maternity wards for safe delivery in the respective hospitals.

### Study population

Pregnant women in the Northern region of Bangladesh.

### Sampling design

The study followed a cross-sectional design where data were collected by direct interviews. Before delivery, the participants were selected by simple random sampling and proportion to the estimated load of deliveries, which accounted for 60 % of all deliveries during the survey period. Most of the questions were close-ended and the answers chosen by the respondents were indicated by the tic mark. The response rate was 100 %.

### Measurement of variables

#### Dependent variables

The dependent variables considered as (i) the types of delivery coded as dichotomous (caesarean = 1, non-caesarean = 0) and (ii) duration of time (that is, after marriage to getting one child or from previous child to current child) to event (that is, mode of delivery).

#### Independent variables

The maternal variables included prolonged labour (more than 12 h), fetal distress (it is commonly used to describe fetal hypoxia that is low oxygen levels in the fetus, which can result in fetal damage if the fetus is not promptly delivered), previous c-section, breathing difficulty, child aborted around delivery, multiple births; head circumference, length and weight of babies.

For the analysis of data, the category related to prolonged labour, fetal distress, previous C-section, breathing difficulty, child aborted around delivery and multiple births were assessed as yes or no. The head circumference of newborns was classified into two categories: <32 cm and more than 32 cm. The length and weight of baby were categorized into: <45 cm or more than 45 cm and <2.5 kg or more than 2.5 kg respectively. The socio-demographic variables included maternal age at birth, age at marriage, parity (order of birth), and maternal educational level. Maternal age was categorized into four broad groups (years): <20, 20–24, 25–29 and more than 30. The age at marriage was classified into three categories: <18 years, 18–22 years and 23 years and above. The parity was divided into three groups: 1, 2, and ≥ 3. Education status is the highest level of schooling attained, measured as primary and below (0–5 years), secondary (6–10 years) and higher (11 years and above). Place of residence and duration of taking balance diet (it refers to milk, fish, egg, fruit and vegetables that contains adequate amounts of all the necessary nutrients required for healthy growth and activity and those diets were taken a woman in pregnancy period) were also considered as the other related variables in the study. Additionally, place of residence was classified as rural verses urban and duration of taking balance diet was measured as a categorical variable: often, once a week and rarely.

### Statistical analysis

An initial bivariate analysis was performed to identify significant associations between types of delivery (caesarean vs. non-caesarean) and a series of independent variables. Dichotomous variables were analysed by the *χ*^2^ test or Fisher exact test, where appropriate. To determine the risk factors which are associated with the C-section, based on the different criteria, two multivariate techniques were used. They are logistic regression model and Cox proportional hazard model. Logistic Regression and Cox proportional hazard models are the most frequently used for analysing data in epidemiological and clinical studies [38]. The logistic regression is analogous to multiple linear regressions where the dependent measure is dichotomous in nature (coded by the values 0 and 1); whereas the Cox proportional regression model assumes that the effects of the predictor variables (names of variables that we expect to predict survival time) are constant over time. For both techniques, maternal, socio-demographic and other relevant variables were treated as independent variables, while the dependent variables were already mentioned in the above section. The most influential risk factors were estimated separately for overall, public and private hospital by stepwise selection. The value of *P* < 0.05 was considered statistically significant. Finally, to identify and measure the risk factors for caesarean delivery, that is, how well the model fits the data, Akaike Information Criterion (AIC) is used. Generally, the AIC formula is −2 log(L) + 2 k, where, L is the maximized value of the likelihood function for the estimated model and k is the number of parameters in the statistical model. Lower AIC indicates a better likelihood.

### Ethical clearance

We obtained informed verbal consent from the respondents before conducting the interview.

The study was approved by the ethical board and research review committee of the Dept. of Population Science & Human Resource Development, University of Rajshahi, Bangladesh.

## Results

Patient characteristics and significant variables are listed in Table 1. The sample comprised of 1142 mothers with the aggregate caesarean section (C-section) rate among the participants being 57.09 %. The C-section rate in the public hospital was 30.28 % (*n* = 199), while the C-section rate in private hospital was 93.47 % (*n* = 453). Caesarean rates varied by level of maternal complications. The significant rate was highest among women having previous C-section (94.8 %). Similarly, the rate was highest among women with higher education level (76.8 %, compared to 44.8 % for mothers with primary and below level of education) followed by higher maternal ages (30 years and above) as compared to lower age groups (less than 20 years). The same pattern was also observed in age at marriage. Residence and nutritional status were among the factors associated with the likelihood of having C-section. C-section deliveries were of low frequency in urban areas as compared to rural areas. The highest Caesarean rate was observed for those who rarely take a balanced diet (76.0 %). Out of 15 variables examined, eight were statistically significant while the remaining eight were statistically not associated with the type of delivery.

**Table 1:** Percentage distributions of maternal, socio-demographic and other characteristics by type of delivery and their significance level in northern region of Bangladesh

Selected variables	Delivery type				*P*-Value
	Caesarean delivery		Non-Caesarean delivery		
	N	%	N	%	
Fetal Distress					0.829
No	588	57.2	440	42.8	
Yes	64	56.1	50	43.9	
Previous C-Section					<0.001
No	561	53.6	485	46.4	
Yes	91	94.8	5	05.2	
Multiple Birth					0.945
No	643	57.1	483	42.9	
Yes	9	56.3	7	43.8	
Pregnancy-Induced Breathing Difficulty					0.993
No	612	57.1	460	42.9	
Yes	40	57.1	30	42.9	
Prolonged Labour					<0.001
No	541	70.0	232	30.0	
Yes	111	30.1	258	69.9	
Mother’s Education					<0.001
Primary and below	147	44.8	181	55.2	
Secondary	310	55.4	250	44.6	
Higher	195	76.8	59	23.2	
Mother’s Age: years					<0.001
<20	185	44.5	231	55.5	
20–24	160	55.2	130	44.8	
25–29	198	69.0	89	31.0	
30+	109	73.2	40	26.8	
Age at Marriage: years					<0.001
<18	344	50.3	340	49.7	
18–22	188	61.2	119	38.8	
23+	120	79.5	31	20.5	
Order of Birth					0.062
1	369	54.7	306	45.3	
2	199	62.6	119	37.4	
3+	84	56.4	65	43.6	
Length of Baby: cm					0.029
<45	457	55.1	372	44.9	
45+	195	62.3	118	37.7	
Weight of Baby: kg					0.894
<2.5	214	57.4	159	42.6	
2.5+	438	57.0	331	43.0	
Head Circumferences: cm					0.180
<32	486	56.0	382	44.0	
32+	166	60.6	108	39.4	
Residence					<0.001
Rural	244	67.6	117	32.4	
Urban	408	52.2	373	47.8	
Ever had a Child Aborted					0.817
No	631	57.2	473	42.8	
Yes	21	55.3	17	44.7	
Duration of Taking Balanced Diet					<0.001
Often	363	51.2	346	48.8	
Once a week	74	49.3	76	50.7	
Rarely	215	76.0	68	24.0	

**Fig. 1:**
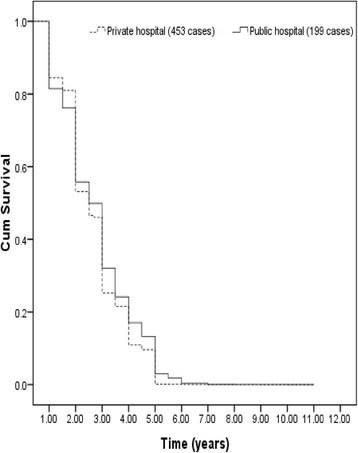
Survival function at mean of covariates (mode of delivery: caesarean)

Figure 1 displays the survival for mean values for selected covariates in the Cox model over time by health facilities. The survival curve represents the probability of mothers who have delivery by caesarean at any given time. During the period of below 2 years, the probability of getting first child from the women in private hospital is greater than those who delivered by caesarean in public hospital. Figure 1 also shows that the rate of caesarean cases over time is relatively constant and approximately below 1 % after the duration time of 6 years and above.

The adjusted odds ratios (ORs) and hazard ratios (HRs) (with 95 % confidence intervals) for a C-section are shown in Tables 2, 3 and 4 at overall, private and public hospitals respectively. As shown in Table 2 within overall delivery cases, the binary logistic regression of multivariate analysis indicated that the risk of having previous C-section (OR = 20.18, CI = 10.46–25.58), prolonged labour (OR = 0.17, CI = 0.12–0.23), higher educational level (OR = 2.68, CI = 1.58–4.54), mother’s age > 25 years (OR = 2.74, CI = 1.58–4.72), lower order of birth (OR = 0.74 CI = 0.49–1.12), length of baby > 45 cm (OR = 1.45, CI = 1.04–2.02), and irregular intake of balance diet (OR = 1.87 CI = 1.24–2.81) significantly predict C-section delivery. The odds ratio 20.18 indicated that the odds of being C-section to the women were seen 20.18 times greater in previous C-section as compared to those were not in previous C-section. The others odds ratio can be explained in the same way. In a Cox’s regression model, the multivariate analysis indicated that fetal distress, previous C-section, mother’s age, age at marriage and lower order of birth were significantly independent risk factors for C-section. The risk of having fetal distress and previous C-section had a higher risk as compared to those who did not. The hazard ratios of C-section for older mothers were higher than their younger counterparts. Women who married at the age of 18–22 years had a lower risk of C-section as compared to women who married at an early age (<18 years) and married at an older age (23 years and above). Similarly, increased parity (order of birth) had a lower risk as compared to lower parity (first order of birth) for C-section.

**Table 2:** Logistic and Cox’s regression results of the effects of selected characteristics on C-section: Overall cases

Selected variables	Results of logistic regression analysis		Results of Cox’s regression analysis	
	Odds ratio [Exp (β)]	95 % CI	Hazard ratio [Exp (β)]	95 % CI
Fetal Distress				
(No)	1.00		1.00	
Yes	1.08	0.67–1.76	1.01^*^	0.52–1.51
Previous C-section				
(No)	1.00		1.00	
Yes	20.18^*^	10.46–25.58	9.80^*^	7.11–12.36
Multiple Birth				
(No)	1.00		1.00	
Yes	1.12	0.35–3.58	1.01	0.54–2.78
Pregnancy-Induced Breathing Difficulty				
(No)	1.00		1.00	
Yes	1.06	0.55–2.03	1.02	0.45–2.35
Prolonged Labour				
(No)	1.00		1.00	
Yes	0.17^*^	0.12–0.23	0.95	0.45–1.89
Mother’s Education				
(Primary and below)	1.00		1.00	
Secondary	2.19^*^	1.55–3.11	1.15	0.57–2.47
Higher	2.68^*^	1.58–4.54	1.53	0.78–2.41
Mother’s Age: years				
(<20)	1.00		1.00	
20–24	1.39	0.92–2.11	1.12^*^	0.75–2.31
25–29	2.74^*^	1.58–4.72	1.48^*^	0.85–2.59
30+	5.07^*^	2.31–11.12	2.05^*^	1.02–3.52
Age at Marriage: years				
(<18)	1.00		1.00	
18–22	0.93	0.64–1.35	0.57^*^	0.31–1.95
23+	1.06	0.54–2.05	1.01^*^	0.54–2.01
Order of Birth				
(1)	1.00		1.00	
2	0.74^*^	0.49–1.12	0.43^*^	0.16–1.57
3+	0.33	0.17–0.64	0.28	0.07–1.47
Length of Baby: cm				
(<45)	1.00		1.00	
45+	1.45^*^	1.04–2.02	1.07	0.45–2.07
Weight of Baby: kg				
(<2.5)	1.00		1.00	
2.5+	0.74	0.54–1.02	0.49	0.17–1.31
Head Circumferences: cm				
(<32)	1.000		1.00	
32+	1.084	0.76–1.54	1.02	0.57–1.89
Residence				
(Rural)	1.00		1.00	
Urban	0.854	0.61–1.91	0.51	0.28–1.18
Ever had a Child Aborted				
(No)	1.00		1.00	
Yes	0.57	0.25–1.33	0.34	0.16–1.51
Duration of Taking Balance Diet				
(Often)	1.00		1.00	
Once a week	1.45^*^	0.95–2.22	1.20	0.54–2.22
Rarely	1.87^*^	1.24–2.81	1.45	0.69–2.07
Intercept	−0.25		−2 log likelihood	13765.19
−2 log likelihood	2997.81		Modelchi-square	398.94^*^
Cox &Snell R^2^	0.54		Degrees of freedom	21
Nagelkerke R^2^	0.57			
AIC	3031.82		AIC	13799.19

^*^ *P* < 0.05, significant risk factors in the model; CI = confidence interval; parentheses indicate the reference categories

**Table 3:** Logistic and Cox’s regression results of the effects of selected characteristics on C-section: Private hospitals

Selected variables	Private hospitals			
	Odds ratio [Exp (β)]	95 % CI	Hazard ratio [Exp (β)]	95 % CI
Fetal Distress				
(No)	1.00		1.00	
Yes	1.32	1.04–2.51	1.03	0.82–1.71
Previous C-section				
(No)	1.00		1.00	
Yes	6.72	3.48–12.05	2.21	1.01–4.03
Multiple Birth				
(No)	1.00		1.00	
Yes	1.32	0.89–2.91	1.01	0.46–2.27
Pregnancy-Induced Breathing Difficulty				
(No)	1.00		1.00	
Yes	1.25	0.98–2.03	1.13	0.52–2.47
Prolonged Labour				
(No)	1.00		1.00	
Yes	0.02^*^	0.01–0.08	0.82	0.28–1.48
Mother’s Education				
(Primary and below)	1.00		1.00	
Secondary	0.49	0.13–1.48	0.24	0.07–1.41
Higher	0.71	0.38–1.93	0.49	0.09–1.78
Mother’s Age: years				
(<20)	1.00		1.00	
20–24	1.01	0.22–2.57	1.01^*^	0.68–2.14
25–29	1.94	0.38–3.58	1.21^*^	0.75–3.15
30+	4.69	0.54–11.01	2.04^*^	1.30–4.21
Age at Marriage: years				
(<18)	1.00		1.00	
18–22	1.00	0.22–3.57	1.52^*^	1.03–2.23
23+	1.05	0.23–5.89	2.14^*^	1.14–3.24
Order of Birth				
(1)	1.00		1.00	
2	0.52^*^	0.16–1.83	0.27^*^	0.05–1.20
3+	0.13	0.03–1.84	0.12^*^	0.04–1.05
Length of Baby’s: cm				
(<45)	1.00		1.00	
45+	0.11	0.03–0.38	0.18	0.08–1.48
Weight of Baby’s: kg				
(<2.5)	1.00		1.00	
2.5+	2.26	0.58–6.32	1.99	0.59–2.81
Head Circumferences: cm				
(<32)	1.00		1.00	
32+	0.56	0.19–1.45	0.38	0.15–1.38
Residence				
(Rural)	1.00		1.00	
Urban	4.60^*^	1.27–12.10	1.93	0.47–2.41
Ever had a Child Aborted				
(No)	1.00		1.00	
Yes	0.34	0.03–1.83	0.23	0.05–1.12
Duration of Taking Balance Diet				
(Often)	1.00		1.00	
Once a week	2.45	0.65–9.63	1.28	0.59–2.21
Rarely	8.23	1.18–14.32	3.31	1.42–5.63
Intercept	4.38		−2 log likelihood	5022.35
−2 log likelihood	1123.01		Model chi-square	189.47^*^
Cox &Snell R^2^	0.51		Degrees of freedom	21
Nagelkerke R^2^	0.53			
AIC	1157.01		AIC	5056.35

^*^ *P* < 0.05, significant risk factors in the model; CI = confidence interval; parentheses indicate the reference categories

**Table 4:** Logistic and Cox’s regression results of the effects of selected characteristics on C-section: Public hospitals

Selected variables	Public hospitals			
	Odds ratio [Exp (β)]	95 % CI	Hazard ratio [Exp (β)]	95 % CI
Fetal Distress				
(No)	1.00		1.00	
Yes	1.57	0.82–2.11	1.23^*^	0.08–2.17
Previous C-section				
(No)	1.00		1.00	
Yes	8.98^*^	5.21–10.51	3.75	1.25–5.36
Multiple Birth				
(No)	1.00		1.00	
Yes	1.59	0.79–2.74	1.30	0.78–2.58
Pregnancy-Induced Breathing Difficulty				
(No)	1.00		1.00	
Yes	1.52	0.78–2.01	1.20	0.55–1.84
Prolonged Labour				
(No)	1.00		1.00	
Yes	0.20^*^	0.11–0.28	0.48	0.07–1.62
Mother’s Education				
(Primary and below)	1.00		1.00	
Secondary	1.65^*^	1.14–2.85	1.27	0.72–2.14
Higher	1.81	0.93–4.38	1.46	0.24–2.36
Mother’s Age: years				
(<20)	1.00		1.00	
20–24	1.97	1.15–3.42	1.35^*^	1.02–2.14
25–29	2.79^*^	1.23–5.36	2.15^*^	1.28–3.20
30+	2.96^*^	0.94–7.32	2.37^*^	1.32–4.17
Age at Marriage: years				
(<18)	1.00		1.00	
18–22	0.89	0.48–1.35	0.51^*^	0.24–1.50
23+	0.88	0.34–2.23	0.66^*^	0.41–1.62
Order of Birth				
(1)	1.00		1.00	
2	0.71	0.46–1.34	0.55^*^	0.22–1.25
3+	0.29^*^	0.19–1.01	0.22^*^	0.09–1.06
Length of Baby’s: cm				
(<45)	1.00		1.00	
45+	1.49^*^	0.95–2.47	1.22	0.16–3.29
Weight of Baby’s: kg				
(<2.5)	1.00		1.00	
2.5+	0.75	0.46–1.12	0.65	0.13–1.81
Head Circumferences: cm				
(<32)	1.00		1.00	-
32+	0.80	0.47–1.27	0.63	0.082
Residence				
(Rural)	1.00		1.00	
Urban	0.82	0.48–1.23	0.78	0.15–1.08
Ever had a Child Aborted				
(No)	1.00		1.00	
Yes	0.48	0.16–1.66	0.31	0.17–1.11
Duration of Taking Balance Diet				
(Often)	1.00		1.00	
Once a week	1.44^*^	0.82–2.59	1.11	0.41–2.31
Rarely	1.73	0.98–2.83	1.42	0.71–3.21
Intercept	−1.19		−2 log likelihood	7196.24
−2 log likelihood	1665.89		Model chi-square	223.72^*^
Cox &Snell R2	0.52		Degrees of freedom	21
Nagelkerke R2	0.55			
AIC	1699.89		AIC	7230.24

^*^ *P* < 0.05, significant risk factors in the model; CI = confidence interval; parentheses indicate the reference categories

To examine the Caesarean delivery with associated risk factors by types of health facilities, separate models were constructed for deliveries in private and public hospitals (Tables 3 and 4). Based on the results of logistic regression alone, it was found that women who have related complications around delivery (previous C-section, prolonged labour) and delivered in public hospitals tend to have higher risk of C-section than those who delivered in private hospitals. In public hospitals, the highest odds ratios for caesarean delivery were seen in women aged 30 years and above (OR = 2.96, CI = 0.94–7.32) as compared to those aged 25 years and below. Similarly, first and second-born babies had higher odds of being delivered by C-section (OR = 0.52, CI = 0.16–1.83) as compared to third or above for deliveries occurring in private hospitals. For the length of baby, compared between the two facilities, it is found that this determinant factor was also less important in public hospitals as compared to private hospital. By comparing the place of delivery, it was a significant determinant of C-section for women delivering in private hospitals, with the strongest risk shown for women residing in urban areas. Finally, a C-section was 1.73 times more likely to occur in public hospitals to women who rarely take a balanced diet. Conversely, to determine independent risk factors for survival time, the Cox’s regression model showed that maternal age, age at marriage and parity were only statistically significant with C-section in both health facilities.

To identify the best regression model for caesarean delivery, we carried out a stepwise regression analysis on the variables in Table 2. In the overall and different hospitals, the most influential significant variables are listed in Tables 5 and 6 respectively. By the stepwise selection, the logistic and Cox’s regression analysis reveals that seven and five remained significant independent risk factors to predict which patients were at highest risk for caesarean delivery. The most common factors were previous C-section, mother’s age and order of birth in both analyses (Table 5). From the different health facilities in Table 6, the logistic regression analysis indicated in a stepwise manner the following risk factors: prolonged labour, length of baby more than 45 cm, urban residence and lower birth order were the most significant determinants of caesarean section in private hospitals, while for public hospitals prolonged labour, previous C-section, and higher mother’s educational level were the most important risk factors for determinants of caesarean delivery in Northern Region of Bangladesh. In a foregoing study using the Cox’s regression model by stepwise method, it is also found that mother’s age, age at marriage and order of birth were the common most influential variables among the selected variables in private and public patients. In addition, Table 2, 3 and 4 also shows that the values AIC is lower in parametric (logistic) model as compare to semi parametric (Cox’s) model. Therefore, the results indicated that the logistic model is the most efficient than Cox’s model for determinant the risk factors for caesarean delivery in multivariable analysis.

**Table 5:** Stepwise regression results of the effects of selected characteristics on C-section: Overall cases

Results of logistic regression by stepwise selection		
Most influential variables among selected variables	Odds ratio [Exp (β)]	95 % CI
Prolonged Labour		
(No)	1.00	
Yes	0.17^*^	0.12–0.23
Previous C-section		
(No)	1.00	
Yes	20.53^*^	10.23–24.92
Mother’s Education		
(Primary and below)	1.00	
Secondary	2.04^*^	1.49–3.01
Higher	2.50^*^	1.48–4.31
Mother’s Age: years		
(<20)	1.00	
20–24	1.35	0.82–1.82
25–29	2.85^*^	1.42–4.12
30+	5.76^*^	2.12–12.41
Order of Birth		
(1)	1.00	
2	0.70	0.48–1.01
3+	0.31^*^	0.12–0.58
Duration of Taking Balance Diet		
(Often)	1.00	
One day per week	1.50^*^	1.10–2.52
Rarely	1.87^*^	1.32–2.85
Length of Baby’s: cm		
(<45)	1.00	
45+	1.46^*^	1.11–2.24
Constant	0.66^*^	0.23–1.57
Results of Cox’s regression by stepwise selection		
Most influential variables among selected variables	Hazard ratio [Exp (β)]	95 % CI
Mother’s Age: years		
(<20)	1.00	
20–24	1.32^*^	1.10–3.23
25–29	1.55^*^	1.21–2.51
30+	2.25^*^	1.25–3.35
Age at Marriage: years		
(<18)	1.00	
18–22	0.69^*^	0.24–1.87
23+	1.10^*^	0.54–2.56
Order of Birth		
(1)	1.00	
2	0.40^*^	0.21–1.36
3+	0.34^*^	0.16–1.53
Fetal Distress		
(No)	1.00	
Yes	1.21^*^	0.69–2.14
Abdominal Operation		
(No)	1.00	
Yes	10.20^*^	3.28–18.62

^*^ *P* < 0.05, significant risk factors in the model; CI = confidence interval; parentheses indicate the reference categories

**Table 6:** Stepwise regression results of the effects of selected characteristics on C-section: Private & Public hospitals

Results of logistic regression by stepwise selection					
Most influential variables among selected variables	Private hospital		Most influential variables among selected variables	Public hospital	
	Odds ratio [Exp (β)]	95 % CI		Odds ratio [Exp (β)]	95 % CI
Prolonged Labour			Prolonged Labour		
(No)	1.00		(No)	1.00	
Yes	0.03^*^	0.01–0.08	Yes	0.21^*^	0.11–0.27
Length of Baby: cm			Previous C-section		
(<45)	1.00		(No)	1.000	
45+	0.17^*^	0.03–0.41	Yes	7.74^*^	4.12–9.36
Residence			Mother’s Education		
(Rural)	1.00		(Primary and below)	1.00	
Urban	4.07^*^	1.31–10.11	Secondary	1.43	1.10–2.73
			Higher	2.59^*^	1.82–5.93
Order of Birth					
(1)	1.00				
2	0.89^*^	0.23–1.21	Constant	0.36^*^	0.12–1.67
3+	0.23^*^	0.12–1.54			
Constant	8.64^*^	2.54–16.82			
Results of Cox’s regression by stepwise selection					
Most influential variables among selected variables	Private hospital		Most influential variables among selected variables	Public hospital	
	Hazard ratio [Exp (β)]	95 % CI		Hazard ratio [Exp (β)]	95 % CI
Mother’s Age: years			Mother’s Age: years		
(<20)	1.00		(<20)	1.00	
20–24	1.04^*^	0.57–2.14	20–24	1.37^*^	0.65–2.36
25–29	1.31^*^	0.45–2.21	25–29	2.17^*^	1.15–3.87
30+	2.25^*^	1.20–4.12	30+	2.57^*^	1.36–4.13
Age at Marriage: years			Age at Marriage: years		
(<18)	1.00		(<18)	1.00	
18–22	1.67^*^	1.02–3.25	18–22	0.61^*^	0.32–1.19
23+	2.35^*^	1.26–4.36	23+	1.21^*^	0.49–2.58
Order of Birth			Order of Birth		
(1)	1.00		(1)	1.00	
2	0.38^*^	0.04–1.13	2	0.65^*^	0.13–1.21
3+	0.23^*^	0.11–1.10	3+	0.41^*^	0.18–1.20
Fetal Distress			Fetal Distress		
(No)	Not found		(No)	1.00	
Yes			Yes	1.51^*^	0.84–3.10

^*^ *P* < 0.05, significant risk factors in the model; CI = confidence interval; parentheses indicate the reference categories

## Discussion

The findings of this study provide us an insight into the impact of maternal, socio-demographic and relevant factors on C-section in the northern region of Bangladesh. The analysis of the C-section deliveries for the private and public hospitals substantiates this concern. The rate of C-section was higher in private hospitals as compared to public hospitals. Past studies in different countries found that the rate of caesarean delivery in private hospitals is also higher than public hospitals [36, 37]. It seems that the private practice of the doctors and the financial motive of the private hospitals may be playing some important role in determining the caesarean rates. This statement is supported by the previous studies [37]. The result from the logistic regression analysis showed that previous C-section, prolonged labour (more than 12 h), maternal education level, mother’s age of more than 25 years, low birth order, length of baby more than 45 cm and irregular intake of a balanced diet were important determinants of C-section. Conversely, two newly independent risk factors (fetal distress and age at marriage) were also found to determinants of C-section by the Cox’s regression model. Furthermore, the association of these determinants with C-section varied by the different health facilities. By the stepwise selection in logistic regression analysis, we confirmed that demographic characteristics such as length of baby, place of residence and order of birth were more important in private facilities whereas mothers complication such as prolonged labour, previous C-section were more significant determinants in public facilities. Moreover, the Cox’s model found that only one factor which is included in mothers’ complication as fetal distress was independent risk factor for C-section in public facilities. Therefore, as shown in these findings, we have expected the rate of C-section will be higher in public patients than in private patients but the observed result shows the inverse.

In the multivariate analysis, educational level, maternal age and parity were found to be the significant non-clinical factors as the ones being the best efficient models in the logistic model. Our results also confirmed by other studies [38, 39]. The findings of the present study may indicate that educated women tend to delay giving birth, thus increasing their likelihood of having C-section. In the previous study, it was found that mother’s education is a proxy of socio-economic variable and it is associated with C-section [40]. In 2001, Ecker et al. [14] cited changes in the childbearing population as a significant cause of the increase of Caesarean birth rates. It is also established that age of mother is closely related to C-section [40]. Nassar & Sullivan [45] suggested that age and parity (order of birth) alone account for most demographic changes because there is a high primary caesarean rate for first birth to women 30 years age and older. Mothers with low birth order who undergo C-section, explained that the choice were made mainly because of their greater risk of pregnancy and delivery-related complications [37, 43, 44]. Therefore, it has been suggested that delivery by caesarean birth is a complicated health issue on a country level and also a global perspective. In addition, place of residence is one of the most important factors in determining whether to perform a C-section in private or public hospital, which is consistent with the findings of other studies [41, 44] have also found that there is a strong association between C-section and place of residence. It seems that women residing in urban areas of the northern region were more likely to undergo C-section in private hospitals. This indicates the importance of social status in determining the type of delivery and also pertains to issues related to disparities in the distribution of health facilities in the country with respect to several studies [45]. Furthermore, numerous socio-economic and cultural factors influence the decision on pattern of feeding and balance diet that may influence the type of delivery. As a point of view, duration of taking balanced diet was considered as an independent variable and the study found that irregular intake of a balanced diet is a significant determinant for caesarean delivery from logistic regression analysis. As also previously mentioned, the significant non-clinical factor found in this study was age at marriage. Therefore, it may indicate that adding more proteins, carbohydrate, vitamins in daily intake will be more beneficial for pregnant women to avoid C-section and decreasing late marriage of the study population for C-section.

## Conclusion

The above discussion leads to the conclusion that delivery by C-section is a complicated health issue. Efforts to reduce C-section birth in developing countries like the northern region of Bangladesh will require a comprehensive approach to address patients’ variables, caregiver practices and hospital policies. In order to address the reduction of caesarean rate in the northern region, significant factors such as previous C-section, prolonged labour, maternal educational level, age at marriage, mother’s age of more than 25 years, low birth order, length of baby more than 45 cm and irregular intake of a balanced diet can be considered to be predictors for C-section. Finally, from the statistical point of view, this study also suggests that these factors may influence the health-seeking behavior of women. Additionally, for determinants of risk factors, the evaluation criteria on AIC, this study imply that logistic regression model can be lead to more precise results as an alternative for the Cox model. Thus, the following steps may be recommended in view of the observed findings:

i. In the study we found that the rate of caesarean delivery is lower in public hospitals than private hospitals. Therefore, medical audit, quality assessment and supportive supervision should be considered to improve the quality of care in a private hospital that is likely to minimize C-section rate.
ii. The result also shows that less than 19 years and more than 25 years old of mothers age are at higher pregnancy risks for C-section. Thus, age group 20 to 24 should be safer for normal delivery. However, future research should review maternal age when examining predictors of caesarean birth.
iii. Encouraging pregnant women to take a balanced and nutritional diet may be beneficial.
iv. Health awareness and educational programs should be given to focus on educating women, on appropriate delivery types when their health and specific status will be known.
v. Provide complete and reliable information to the mothers so that they do not opt for C-section in a state of panic or ignorance.
vi. Universities and schools who educate health team (doctors, midwives, and nurses) offer topic that directly deal with this subject.
vii. Further research at the national level with other medical procedures is highly recommended to figure out the extent of this problem in Northern region of Bangladesh.
viii. Moreover, Government should be given more attention to monitor hospital data and corresponding strategies.
